# Distinguishing Features of Cetuximab and Panitumumab in Colorectal Cancer and Other Solid Tumors

**DOI:** 10.3389/fonc.2019.00849

**Published:** 2019-09-20

**Authors:** Jesús García-Foncillas, Yu Sunakawa, Dan Aderka, Zev Wainberg, Philippe Ronga, Pauline Witzler, Sebastian Stintzing

**Affiliations:** ^1^Cancer Institute, University Hospital Fundacion Jimenez Diaz, Autonomous University of Madrid, Madrid, Spain; ^2^Department of Clinical Oncology, St. Marianna University School of Medicine, Kawasaki, Japan; ^3^Chaim Sheba Medical Center, Ramat Gan, Israel; ^4^David Geffen School of Medicine at University of California, Los Angeles, CA, United States; ^5^Merck Healthcare KGaA, Darmstadt, Germany; ^6^Department of Hematology, Oncology, and Tumor Immunology (CCM) Charité Universitaetsmedizin, Berlin, Germany

**Keywords:** colorectal cancer, cetuximab, panitumumab, FOLFOX, FOLFIRI, antibody-dependent cell-mediated cytotoxicity

## Abstract

Cetuximab and panitumumab are two distinct monoclonal antibodies (mAbs) targeting the epidermal growth factor receptor (EGFR), and both are widely used in combination with chemotherapy or as monotherapy to treat patients with *RAS* wild-type metastatic colorectal cancer. Although often considered interchangeable, the two antibodies have different molecular structures and can behave differently in clinically relevant ways. More specifically, as an immunoglobulin (Ig) G1 isotype mAb, cetuximab can elicit immune functions such as antibody-dependent cell-mediated cytotoxicity involving natural killer cells, T-cell recruitment to the tumor, and T-cell priming via dendritic cell maturation. Panitumumab, an IgG2 isotype mAb, does not possess these immune functions. Furthermore, the two antibodies have different binding sites on the EGFR, as evidenced by mutations on the extracellular domain that can confer resistance to one of the two therapeutics or to both. We consider a comparison of the properties of these two antibodies to represent a gap in the literature. We therefore compiled a detailed, evidence-based educational review of the known molecular, clinical, and functional differences between the two antibodies and concluded that they are distinct therapeutic agents that should be considered individually during treatment planning. Available data for one agent can only partly be extrapolated to the other. Looking to the future, the known immune activity of cetuximab may provide a rationale for this antibody as a combination partner with investigational chemotherapy plus immunotherapy regimens for colorectal cancer.

## Introduction

The advent of targeted monoclonal antibodies (mAbs) brought a revolution in the field of oncology. With increased specificity, longer half-lives, and more predictable overall pharmacokinetic and pharmacodynamic behaviors than their small-molecule inhibitor counterparts, mAbs have become key components of standard-of-care treatments for multiple indications. Inevitably, sometimes several approved mAbs against the same target are available, requiring physicians to perform detailed research to understand which mAb is the optimal therapeutic agent for a given patient. In fact, more than half of the approved targeted mAbs in oncology (excluding the new wave of checkpoint inhibitors) are clustered around 5 targets: the epidermal growth factor receptor (EGFR), the human epidermal growth factor receptor 2 (HER2), tumor necrosis factor α, CD20, and vascular endothelial growth factor (VEGF) (1). Indeed, among treatment options for metastatic colorectal cancer (mCRC), in particular, are two anti-EGFR mAbs, cetuximab and panitumumab, currently indicated for the same subgroup of patients, those with *RAS* wild-type (wt) metastatic disease (2, 3). Approximately 40% of patients with CRC will eventually develop metastatic disease (4); per international guidelines, the majority of these patients should undergo *RAS* testing for suitability for an anti-EGFR mAb in combination with oxaliplatin- or irinotecan-based chemotherapy. Thus, clinicians must choose between prescribing cetuximab and panitumumab regularly.

In 2004, cetuximab was approved by both the US FDA and the EMA for use in EGFR-expressing (*K*)*RAS*-unselected chemorefractory mCRC. Panitumumab was approved by the US FDA for use in the same patient population in 2006. In 2007, the EMA rejected the use of panitumumab in an unselected chemorefractory population, but approved the use of panitumumab in a restricted population of *KRAS* exon 2 wt mCRC, and imposed a similar restriction on use of cetuximab in 2008. By 2009, the FDA followed the EMA by restricting use of either anti-EGFR agent to *KRAS* exon 2 wt chemorefractory mCRC patients.

In the first-line setting, panitumumab + CT was approved by the EMA in 2011, based on positive results from the randomized phase 3 PRIME trial. In 2012, cetuximab + CT was approved by the FDA following the phase 3 CRYSTAL trial. In 2013, extended *RAS* testing was required by the FDA and EMA for predicting response to anti-EGFR agents (5).

According to the EU SmPC, cetuximab is currently indicated for EGFR-expressing *RAS* wt mCRC as a monotherapy in patients who have failed oxaliplatin- and irinotecan-based therapy and who are intolerant to irinotecan, in combination with irinotecan-based therapy in any line, and in combination with FOLFOX in first-line. Cetuximab is also indicated for use in SCCHN, both in locally advanced disease (in combination with radiation therapy) and in recurrent/metastatic disease (in combination with platinum-based chemotherapy). Panitumumab is indicated for *RAS* wt mCRC as a monotherapy after failure of fluoropyrimidine-, oxaliplatin-, and irinotecan-containing chemotherapy regimens, in combination with FOLFOX or FOLFIRI in first-line, and in combination with FOLFIRI in second-line mCRC (6, 7).

To date, >480,000 patients with mCRC have received cetuximab-based therapy worldwide, and >240,000 patients with mCRC have been treated with panitumumab-containing therapy (8, 9). Although these two mAbs are considered to be very similar, important biological, molecular, and practical differences exist between them. Thus, there are uncertainties regarding whether they can be considered equivalent and whether it is prudent to ascribe conclusions gleaned from a study of one agent to the other and to pool data on the two in meta-analyses. In this article, we summarize and discuss these differences, primarily within the context of mCRC, but we also describe their differential activity in the treatment of squamous cell carcinomas of the head and neck (SCCHN). We then relate how these differences could impact the potential for anti-EGFR mAbs to be combined with emerging immunotherapies. The goal of this review is to provide a comprehensive discussion of the available data on the two mAbs and to highlight how they are distinct therapeutic agents with individual, clinically relevant properties.

## Mode of Action Against EGFR

Dysregulation in the EGFR signaling pathway has long been associated with pro-oncogenic activities such as increased cell proliferation, reduced apoptosis, and increased angiogenesis and metastatic tendencies (1, 4, 10). The EGFR is activated when one of its many ligands (including the epidermal growth factor [EGF], transforming growth factor α, amphiregulin, or epiregulin) binds the receptor's extracellular domain, resulting in receptor dimerization, conformational change, and tyrosine autophosphorylation (4, 10, 11). Upon receptor binding, downstream signaling cascades including the MAPK/ERK (mitogen-activated protein kinase/extracellular signal-regulated kinase), JAK/STAT (Janus kinase/signal transducers and activators of transcription), and PI3K/Akt (phosphoinositide 3-kinase/protein kinase B) pathways become active. Constitutive activation of these pathways can lead to cancer cell survival and proliferation (1, 4, 5).

Cetuximab and panitumumab both function by binding to the extracellular domain III of the EGFR, thereby preventing ligand binding and locking the EGFR in the autoinhibitory monomeric conformation (1, 4, 11). The antibody-receptor construct is then internalized, ubiquitinated, and either degraded or recycled. This turnover is regulated by the ubiquitin proteasome system (12, 13). Briefly, after activation of the receptor tyrosine kinase through ligand binding and dimerization, the activated receptor is internalized by clathrin-dependent endocytosis and ubiquitinated. This process terminates the tyrosine kinase activity of activated EGFR and regulates the number of receptors expressed on the cell surface. The final step of degradation is performed by the proteasome; however, ubiquitinated receptors can be deubiquitinated by deubiquitinating enzymes and then recycled back to the cell membrane (12). Receptor ubiquitination has been identified as a mechanism of resistance to anti-EGFR therapy (12).

Between 60 and 80% of colorectal tumors overexpress the EGFR; although this characteristic was historically thought to be predictive of response to cetuximab and panitumumab, in more recent years this notion has not held up in practice (1, 4, 14). Alternative explanations for the efficacy of cetuximab and panitumumab in colorectal tumors regardless of EGFR overexpression status focus on the ligands to EGFR and potential dysregulation of the amount of ligands produced and released into the extracellular space (5). Indeed, both cetuximab and panitumumab compete with EGF for its binding site on EGFR. Mutational studies have demonstrated that the two mAbs have different binding sites on EGFR, but the binding epitopes are in close physical proximity and have some key residues in common (15) (Table 1). Panitumumab's binding epitope includes EGFR residues P349, P362, D355, F412, and I438, all of which are individually necessary for ≥50% binding affinity (15). In contrast, binding residues on EGFR critical for cetuximab binding are Q384, Q408, H409, K443, K465, I467, and S468, as well as F352, D355, and P387 (15). D355 is likely a source of competition between the mAbs and EGF because it is within the binding site of all three molecules (15). Notably, panitumumab's binding epitope overlaps with the EGF binding site in two locations (D355 and K443), whereas cetuximab overlaps with EGF's binding site in 5 locations (D355, Q408, H409, K443, and S468).

**Table 1:** Basic comparison of cetuximab and panitumumab.

**Variable**	**Cetuximab**	**Panitumumab**
Approved indications (acc.to EU label)	mCRC: in combination with irinotecan-based chemotherapy in first-line in combination with FOLFOX as a single agent in patients who have failed oxaliplatin- and irinotecan-based therapy and who are intolerant to irinotecan SCCHN: in combination with radiation therapy for LA SCCHN in combination with platinum-based chemotherapy for R/M SCCHN	mCRC: in first-line in combination with FOLFOX or FOLFIRI in second-line in combination with FOLFIRI for patients who have received first-line, fluoropyrimidine-based chemotherapy (excluding irinotecan) as monotherapy after failure of fluoropyrimidine-, oxaliplatin-, and irinotecan-containing chemotherapy regimens
IgG isotype	IgG1	IgG2
Fc	Chimeric (mouse/human)	Human
EGFR binding sites in the EGF-binding pocket	D355, Q408, H409, K443, S468	D355, K443
K_D_	0.39 nM	0.050 nM
Immune activity	NK cell–driven ADCC, CDC	Monocyte/neutrophil-driven ADCC
Registered dose/posology	400 mg/m^2^ initial dose as a 120-min IV infusion, followed by 250 mg/m^2^ weekly as a 60-min IV infusion	6 mg/kg every 2 weeks as an IV infusion over 60 min (≤ 1,000 mg) or 90 min (>1,000 mg)

*ADCC, antibody-dependent cell-mediated cytotoxicity; CDC, complement-mediated cytotoxicity; CRC, colorectal cancer; EGFR, epidermal growth factor receptor; FOLFOX, oxaliplatin, leucovorin, and fluorouracil; Ig, immunoglobulin; IV, intravenous; K_D_, dissociation constant; LA, locally advanced; NK, natural killer; R/M, recurrent and/or metastatic; SCCHN, squamous cell carcinoma of the head and neck*.

Furthermore, cetuximab and panitumumab have different binding affinities for EGFR, with dissociation constants (K_D_) of 0.39 nM vs. 0.05 nM, respectively (4). Cetuximab binds EGFR with ~2-fold greater affinity than EGF (16). Panitumumab binds EGFR with an ~8-fold greater affinity than that of cetuximab. However, it is unclear whether this characteristic is favorable. From one standpoint, a higher affinity for EGFR should translate into a greater proportion of mAb-bound EGFR; conversely, however, studies have observed that a K_D_ between 1 and 10 nM is optimal for anti-EGFR mAb tumor targeting, accumulation, and retention (11). Although the K_D_ of cetuximab is closer, neither mAb is within the optimal range. Cetuximab and panitumumab administration schedules are very different from each other (Table 1). Cetuximab is administered based on body surface area, and is usually given as a 400-mg/m^2^ initial dose by a 120-min intravenous (IV) infusion, followed by a weekly dose of 250 mg/m^2^ by 60-min IV infusion (6). However, Q2W doses of 500 mg/m^2^ have been investigated; this dosing schedule is frequently used, and is recommended based on NCCN guidelines but not approved by regulatory authorities (3). Maintenance cetuximab can be administered on the same weekly or Q2W schedule (17) and treatment with cetuximab is recommended to be given until progression of disease (6). Indeed, in pharmacokinetic studies, a 250-mg/m^2^ weekly cetuximab dose has a mean half-life of 4.19 days and a minimum recorded mean concentration of 49.6 μg/mL (17). By comparison, panitumumab is administered by weight at a dose of 6 mg/kg every 2 weeks; a 60-min infusion time is recommended for total doses ≤ 1,000 mg, and a 90-min infusion time is recommended for total doses > 1,000 mg (7). At this administration schedule, panitumumab's mean half-life is 7.5 days, with a minimum recorded mean serum concentration of 39 μg/mL (18). Studies have indicated that it takes 3 infusions of panitumumab to reach steady state (19), although similar information has not been published for cetuximab. Overall, administration of cetuximab and panitumumab per their standard schedules results in comparable pharmacokinetic behaviors and overall drug exposures. One final structural difference between the two mAbs is found in their respective backbones. Panitumumab is a human mAb and cetuximab is a mouse/human chimeric mAb. Although this distinction can sometimes lead to differences in the rates of infusion-related reactions between the two agents, these can be managed with the appropriate pre-medication prior to infusion.

## Molecular Structure and Associated Immune Activity

**Figure 1:**
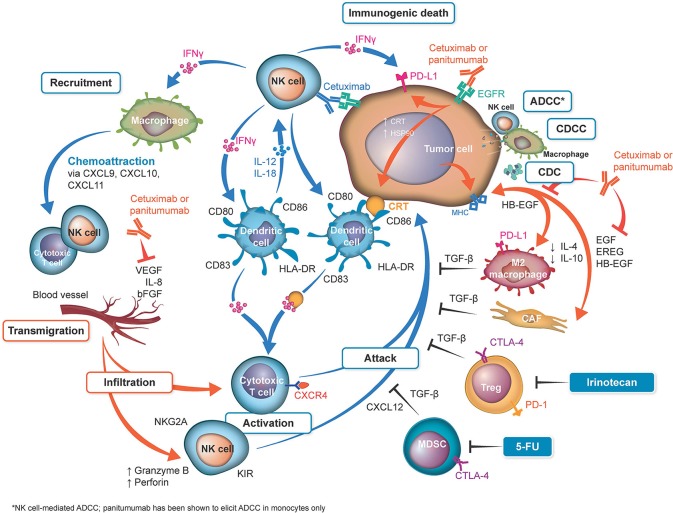
Overview of differences in immune activation with cetuximab and panitumumab. **Shown in orange:** sites of activation by both anti–epidermal growth factor receptor (EGFR) monoclonal antibodies (mAbs). Both anti-EGFR mAbs neutralize the cross talk between the cancer cells and M2 monocytes and cancer-associated fibroblasts (CAFs) by neutralization of EGFR ligands. On the basis that cetuximab and panitumumab may have identical effects, from a mechanistic point of view, both antibodies reduce vascular endothelial growth factor (VEGF) production (20, 21). Cetuximab can upregulate calreticulin (CRT), heat shock protein (HSP) 90, and major histocompatibility complex (MHC) (22, 23), which may be theoretically upregulated by panitumumab (not reported). **Shown in blue:** sites activated by cetuximab. Natural killer (NK) cells are activated by their binding to the cetuximab loaded onto EGFR (22, 24, 25). The released interferon γ (IFN-γ) activates dendritic cells (DCs), which further activate the NK cells (26). Cetuximab-induced antibody-dependent cell-mediated cytotoxicity (ADCC), complement-dependent cell-mediated cytotoxicity (CDCC), complement-mediated cytotoxicity (CDC) (27–30), and immunogenic death (31) release tumor antigens, which are captured by the activated DC cells, to be presented to T cells (thus activating them). IFN-γ upregulates programmed cell death 1 ligand 1 (PD-L1) on tumor cells and activates macrophages to release chemoattraction substances for NK cells and T cells (25). Inhibition of the angiogenic factors VEGF, interleukin (IL) 8, and fibroblast growth factor (FGF) can be downregulated by both cetuximab and possibly by panitumumab (20, 21). Inhibition of these factors upregulates key homing adhesion molecules for the immune cells (intercellular adhesion molecule 1 [ICAM-1] and vascular cell adhesion protein 1 [VCAM-1]) (32, 33) and downregulates Fas antigen ligand (FasL) expression (34), which would be lethal for T cells. These effects enable the safe transmigration of T cells and NK cells into the tumor microenvironment (35). The T cells activated by DCs loaded with tumor cell antigens are then ready to attack the tumor cells. **Shown in black**: Immune suppressive mechanisms/prevention of the successful attack of activated cytotoxic T cells on tumor cells. These mechanisms include checkpoint inhibitory factors (programmed cell death 1 protein [PD-1], PD-L1, cytotoxic T-lymphocyte protein 4 [CTLA-4]) and TGF-β generated by tumor-associated cells (25). Notably, irinotecan and fluorouracil (5-FU) can eliminate tumor protective cells, such as regulatory T cells (Tregs) and myeloid-derived suppressor cells (MDSCs), from the tumor microenvironment (36, 37), reducing their immune suppressive effects and thus potentially facilitating the T-cell attack. bFGF, basic fibroblast growth factor; EREG, epiregulin; HB-EGF, heparin-binding EGF-like growth factor; HLA, human leukocyte antigen; KIR, killer cell immunoglobulin-like receptor; TGF-β, transforming growth factor β.

One of the most hotly debated topics is the functional implication of the differing immunoglobulin (Ig) G subtypes of cetuximab and panitumumab—namely, that cetuximab is an IgG1 isotype mAb, whereas panitumumab has the IgG2 backbone (Figure 1, Table 1) (4, 38). The two Ig isotypes differ in their ability to mobilize innate and adaptive immune cells against tumor cells (Figure 1, Table 2). For example, it has been demonstrated in preclinical models and *ex vivo* studies that target-bound cetuximab and other IgG1 isotype mAbs (e.g., rituximab, necitumumab, trastuzumab) stimulate natural killer (NK) cell–driven cytotoxicity against tumor cells coated in mAbs via the interaction of the constant region and the CD16 receptor on NK cells (38, 44–47). This antibody-dependent cell-mediated cytotoxicity (ADCC) is specifically carried out by NK cells of the innate immune system against tumor cells, resulting in antigen release into the intratumoral space (16). By secreting cytokines and interferon γ, active NK cells are further able to stimulate dendritic cell (DC) maturation and DC-NK cell cross talk (24, 27, 38) and use increased expression of CD137 to recruit anti-EGFR CD8^+^ cytotoxic T cells to the intratumoral space for additional cell-killing activity (40, 41, 48). In turn, mature DCs can mobilize a number of additional immunogenic processes, including antigen presentation to cytotoxic T cells and further activation of NK cells (24, 27, 38, 48). Collectively, NK cell–mediated ADCC and other immunogenic activity of IgG1 mAbs is thought to contribute to their antitumor activity, provided that sufficient target is available for the mAbs to dually bind to CD16 and their intended epitope (46, 49–51). This sequence of immune events initiated by cetuximab can be viewed as a chain reaction reminding of a domino effect (Figure 1). Furthermore, some clinical evidence has suggested that patients with higher baseline ADCC activity or specific CD16 polymorphisms that increase NK cell–binding affinity might be likelier to experience favorable outcomes with IgG1-based therapy (28, 52–55). By contrast, the Fc region of the IgG2 backbone of panitumumab has very low binding affinity for CD16; thus, panitumumab is unable to induce NK cell–driven ADCC or cytotoxic T-cell tumor infiltration (16, 48), although evidence suggests that panitumumab instead induces some immunostimulatory action via neutrophil-driven ADCC and monocytes (1, 27). However, its immunogenic properties are not considered to actively contribute to panitumumab's antitumor activity (47, 48). A final difference in the immunostimulatory capabilities of IgG1 and IgG2 mAbs concerns the C1 complex of complement, which can be induced by clusters (hexamers) of IgG1 mAbs but has not been shown to be induced to the same degree by IgG2 mAbs (47, 56, 57).

**Table 2:** Cetuximab and panitumumab: differences in immune activation.

**Variable**	**Cetuximab**	**Panitumumab**
**Cetuximab-related immune cytotoxicity**		
ADCC	Yes (27–30)	Activates neutrophil-mediated ADCC and monocytes (1, 27)
CDCC	Yes (29)	–
CDC	Yes (29)	–
**Effects on microenvironment cytokines and MMP**		
Downregulation of IL-8	Yes (20, 21)	Probably
Downregulation of VEGF	Yes^a^ (20, 21)	Yes (20, 21)
Downregulation of bFGF	Yes (20, 21)	Probably
Downregulation of MMP-9	Yes (39)	Probably
**Effects on NK cells**		
NK cell chemoattraction	Yes (35)	No
Increased NK cell infiltration	Yes (31, 35)	No
NK cell activation and HLA expression	Good (22, 24, 25)	No (24)
NK cell activation (CD137 upregulation)	Good (40, 41)	Less (27)
IFN-γ induction by NK cells	Yes (24)	No (24)
Increase in TAP-1 in NK cells	Yes (24)	No (24)
Cross-presentation of tumor antigens by NK cells	Significantly better (27)	No (27)
**Effects on DCS**		
DC maturation (increase in CD80, CD83, CD86, HLA-DR)	Good (23, 24)	No (24)
DC activation	Good (23, 24)	No (24)
Increase in TAP-1 and TAP-2 in DCs (activation)	Yes (24, 42)	No (24)
DC upregulation of MHC class I (MICA)	Yes (24)	Not reported
Enhanced reciprocal DC-NK cell activation/cross talk	Yes (24, 27)	No or significantly reduced (24, 27)
Increased DC phagocytosis	Yes (23)	Not reported
Increase in efficiency of antigen cross-presentation by DCs to T cells	Good (24)	Weak (24)
**Effects on macrophages**		
Macrophage activation	Yes (indirect) (25)	Not expected
**Effect on cytotoxic T cells**		
Increased T-cell chemoattraction	Yes (35)	Not expected
Increased T-cell infiltration	Yes (35)	Not expected
T-cell activation	Yes (24)	Significantly less than cetuximab (24)
**Immune priming effects on tumor cells**		
Upregulation of MHC class I	Yes (24)	Possibly
Immunogenic cell death	Yes (31)	Not reported
**Immune responses induced by cetuximab** **+** **irinotecan combination**		
**Effects on microenvironment cytokines**		
IL-2 increase	Yes (26)	Not reported
IFN-γ increase	Yes (26)	Not reported
IL-12 increase	Yes (26)	Not reported
IL-18 increase	Yes (26)	Not reported
IL-4 decrease	Yes (26)	Not reported
**Effects on immune cells in the TME**		
Increase in circulating NK cells	Yes (43)	Not reported
Increase in circulating DCs	Yes (43)	Not reported
DC activation	Yes (23)	Not reported
Increased DC phagocytosis and trogocytosis	Yes (23)	Not reported
Increase in activated T cells	Yes (43)	Not reported
Increase in central memory cells	Yes (43)	Not reported
Treg elimination	Yes (26)	Not reported
**Immune effects on tumor cells**		
Increase in tumor cell immunogenicity by upregulating calreticulin, HSP 90	Yes (23)	Not reported
Increased immunogenic death	Yes (31)	Not reported
Improved immune “contexture”	Yes (26)	Not reported

a *On the basis that cetuximab and panitumumab may have identical effects, from a mechanistic point of view*.

*ADCC, antibody-dependent cell-mediated cytotoxicity; bFGF, basic fibroblast growth factor; CAF, cancer-associated fibroblasts; CDC, complement-mediated cytotoxicity; CDCC, complement-dependent cell-mediated cytotoxicity; DC, dendritic cell; HLA, human leukocyte antigen; HSP 90, heat shock protein 90; IFN-γ, interferon γ; IL, interleukin; MDSC, myeloid-derived suppressor cell; MHC, major histocompatibility complex; MICA, MHC class I polypeptide-related sequence A; MMP, matrix metalloproteinase; NK, natural killer; TAP, transporter associated with antigen processing; TME, tumor microenvironment; Treg, regulatory T cell; VEGF, vascular endothelial growth factor*.

## Biomarkers of Response, Target Populations, and Therapeutic Resistance

Colorectal cancer is a highly heterogeneous disease (5), characterized by predictive and prognostic mutations (58, 59) as well as a tendency to undergo clonal selection under drug pressure and develop acquired resistance to certain therapies (60–62). For example, as recommended by the international guidelines, both cetuximab and panitumumab are suitable only for patients with *RAS* wt colorectal tumors, with genetic analysis of *KRAS* exon 2 (codons 12, 13), exon 3 (codons 59, 61), exon 4 (codons 117, 146) and *NRAS* exon 2 (codons 12, 13), exon 3 (codons 59, 61), and exon 4 (codons 117, 146) (“*RAS* wt”) (2, 3, 5, 63). Although several early retrospective *RAS* analyses (58, 64) provided evidence supporting testing beyond *KRAS* exon 2 (i.e., extended *RAS* analysis), the retrospective analysis of the PRIME study was the first phase 3 analysis to support refinement of the patient population by *RAS* status and the need for extended *RAS* analyses. In PRIME, panitumumab in combination with FOLFOX4 was shown to have greater benefit in a *RAS* wt-targeted patient population rather than in a patient population identified as *KRAS* wt, compared with FOLFOX4 alone (65). Additional *post hoc* analyses of several phase 3 trials involving cetuximab have also demonstrated improved responses and survival with cetuximab-based therapy with FOLFOX or FOLFIRI in patients with *RAS* wt mCRC compared with patients with *KRAS* wt tumors (66–68). Results from the TAILOR trial, the first phase 3 study to prospectively recruit a *RAS* wt patient population for first-line treatment of mCRC with cetuximab plus chemotherapy (specifically, FOLFOX), further confirmed the survival benefit with cetuximab-based treatment in *RAS* wt mCRC (69). Finally, *KRAS* amplification, although much rarer than and nearly always mutually exclusive with *KRAS* mutations (amplification is present in ~1–2% of cases of mCRC) (5), has been shown to confer resistance to cetuximab and panitumumab and is considered an emerging biomarker by current guidelines (2).

In addition to mutations existing in the predominant cell population of the tumor before treatment, overall resistance to therapy can arise during anti-EGFR therapy, as the drug can inhibit growth of sensitive clones, thereby allowing for expansion of initially rare *RAS*-mutant clones (10, 62). Indeed, there is preclinical and clinical evidence available demonstrating that *RAS* wt tumors can “switch” to *RAS* mutant after anti-EGFR treatment (with either cetuximab or panitumumab) (70), likely because of a significant reduction of the wt clone and an expansion of mutated clones. Finally, recent studies have suggested the possibility of a restoration of responsiveness to cetuximab after the development of resistance to previous cetuximab treatment (71, 72). The prospective CRICKET study, which evaluated third-line re-treatment with cetuximab plus irinotecan after an initial response followed by progression while patients had received the same regimen in the first line, showed that *RAS* wt status in circulating tumor DNA before start of third-line therapy was significantly associated with prolonged progression-free survival (PFS) compared with a *RAS* mutated status (73).

Resistance to anti-EGFR therapy can also be conferred through extracellular domain mutations in the EGFR itself, which have been observed in only EGFR therapy–experienced patients, suggesting that these mutations arise specifically as a mechanism of acquired resistance (13, 60, 74–76). Notably, different mutations in the extracellular domain can dictate resistance only to cetuximab, only to panitumumab, or to both mAbs, owing to their differential binding sites (15). For example, the S492R and S468R mutations in the extracellular domain of the EGFR confer resistance only to cetuximab (13, 75), whereas the G465R mutation that arises in 1 of every 6 patients who receive panitumumab confers resistance to both mAbs (77). Such observations may have implications for planning treatment sequencing, treatment continuation, and maintenance therapy designed to maximize the number of efficacious lines of therapy and the likelihood of response at each stage.

## Clinical Impact of Cetuximab and Panitumumab in Colorectal Cancer

Over the last two decades, cetuximab and panitumumab have been evaluated for efficacy and safety in mCRC in many clinical trials. With approximately half a million patients treated with cetuximab, and close to a quarter of a million treated with panitumumab, the clinical impact of these two mAbs on the disease has been substantial. Currently, the median overall survival (OS) in patients who present with *RAS* wt metastatic disease is usually ≥ 30 months, with hazard ratios (HRs) for survival with first-line cetuximab-based therapy of 0.763 in combination with FOLFOX vs. FOLFOX alone, 0.69 in combination with FOLFIRI vs. FOLFIRI alone, and 0.70 to 0.90 in combination with either doublet chemotherapy vs. bevacizumab plus doublet chemotherapy, according to phase 3 trials (66, 67, 69, 78). Although panitumumab has not been extensively studied in combination with FOLFIRI chemotherapy in the first-line setting, first-line panitumumab plus FOLFOX vs. FOLFOX alone yielded an HR for survival of 0.78 in a retrospective analysis of the RAS wt population of the phase 3 PRIME trial (65). Additionally, a retrospective analysis of the phase 2 PEAK trial yielded an HR for survival of 0.63 with panitumumab plus FOLFOX vs. bevacizumab plus FOLFOX in the population with *RAS* wt disease; however, patient numbers were much lower in this phase 2 study than in the analogous cetuximab phase 3 CALGB/SWOG 80405 and FIRE-3 trials (63, 67, 78).

A full summary of the available first-line data for cetuximab and panitumumab in combination with chemotherapy is presented in Table 3. Notably, however, while cetuximab has been shown to pair well with FOLFIRI, FOLFOX, and FOLFOXIRI (leucovorin, 5-FU, oxaliplatin, and irinotecan) chemotherapy backbones in multiple randomized studies (66, 67, 78, 85, 86, 89), almost all available data for panitumumab in the first-line *RAS* wt setting are in combination with FOLFOX and include only 1 phase 3 and 1 phase 2 study. Evidence for panitumumab plus FOLFIRI in mCRC comes from two studies. The first was a phase 2, single-arm study of panitumumab + FOLFIRI in first-line mCRC, which showed favorable efficacy of the combination in *KRAS* wt vs. *KRAS* mt mCRC (90). The second study was the phase 3 20050181 trial, which administered this combination in the second-line setting in patients with *KRAS* wt mCRC. The phase 3 second-line study reported a significant but modest improvement in PFS compared with FOLFIRI alone (median, 6.7 vs. 4.9 months; HR, 0.82), a trend toward improvement in OS (median, 14.5 vs. 12.5 months; HR, 0.92), and a significant improvement in objective response rate (ORR; 36 vs. 10%) (91). Recently, the phase 2 Gruppo Oncologico del Nord Ovest (GONO) and VOLFI trials provided published evidence for the first-line panitumumab combination with FOLFOXIRI in patients (*N* = 37 and *N* = 96, respectively) with non–liver-limited mCRC (88, 92).

**Table 3:** Clinical impact of cetuximab and panitumumab in *RAS* wt mCRC^*^.

**Study**	**Patients, *n***	**Treatment regimen**	**Median PFS, months**	**Median OS, months**	**ORR, %**
CALGB/SWOG (78–80)	270 vs. 256	Cetuximab + FOLFOX/FOLFIRI vs. bevacizumab + FOLFOX/FOLFIRI	11.4 vs. 11.3 (HR, 1.1 [95% CI, 0.9–1.3]; *P* = 0.31)	32.0 vs. 31.2 (HR, 0.9 [95% CI, 0.7–1.1]; *P =* 0.40)	68.8 vs. 56.0 (*P* < 0.01)
FIRE-3 (67)	199 vs. 201	Cetuximab + FOLFIRI vs. bevacizumab + FOLFIRI	10.3 vs. 10.2 (HR, 0.97 [95% CI 0.78–1.20])	33.1 vs. 25.0 (HR, 0.70 [95% CI, 0.54–0.90])	65.3 vs. 58.7 (OR, 1.33 [95% CI, 0.88–1.99])
CRYSTAL (66)	178 vs. 189	Cetuximab + FOLFIRI vs. FOLFIRI	11.4 vs. 8.4 (HR, 0.56 [95% CI, 0.41–0.76]; *P* < 0.001)	28.4 vs. 20.2 (HR, 0.69 [95% CI, 0.54–0.88]; *P =* 0.0024)	66.3 vs. 38.6 (OR, 3.11 [95% CI, 2.03–4.78]; *P* < 0.001)
COIN (81)	362 vs. 367	Cetuximab + oxaliplatin + fluoropyrimidine vs. oxaliplatin + fluoropyrimidine	8.6 vs. 8.6 (HR, 0.96 [95% CI, 0.82–1.12]; *P* = 0.60)	17.0 vs. 17.9 (HR, 1.04 [95% CI, 0.87–1.23]; *P =* 0.67)	64 vs. 57 (OR, 1.35 [95% CI, 1.00–1.82]; *P =* 0.049)
OPUS (82, 83)	38 vs. 49	Cetuximab + FOLFOX vs. FOLFOX	12.0 vs. 5.8 (HR, 0.53 [95% CI, 0.27–1.04]; *P* = 0.0615)	19.8 vs. 17.8 (HR, 0.94 [95% CI, 0.56–1.56]; *P =* 0.80)	58 vs. 29 (OR, 3.33 [95% CI, 1.36–8.17]; *P =* 0.0084)
TAILOR (69)	193 vs. 200	Cetuximab + FOLFOX vs. FOLFOX	9.2 vs. 7.4 (HR, 0.69 [95% CI, 0.54–0.89]; *P* = 0.004)	20.7 vs. 17.8 (HR, 0.76 [95% CI, 0.61–0.96]; *P =* 0.02)	61.1 vs. 39.5 (OR, 2.41 [95% CI, 1.61–3.61]; *P* < 0.001)
BELIEF (84)	45 vs. 48	Cetuximab + FOLFOX/FOLFIRI vs. FOLFOX/FOLFIRI	9.8 vs. 5.3 (HR, 0.52 [95% CI, 0.33–0.81]; *P* = 0.002)	35.1 vs. 21.7 (HR, 0.44; [95% CI, 0.23–0.83]; *P =* 0.009)	62.2 vs. 29.2
MACBETH (85, 86)	59 vs. 57	Cetuximab + mFOLFOXIRI (with cetuximab maintenance) vs. cetuximab + FOLFOXIRI (with bevacizumab maintenance)	10.1 vs. 9.3 (HR, 0.83 [95% CI, 0.57–1.21])	33.2 vs. 32.2 (HR, 0.92 [95% CI, 0.57–1.47])	71.6% in the entire cohort
PEAK (63)	88 vs. 82	Panitumumab + FOLFOX vs. bevacizumab + FOLFOX	13.0 vs. 9.5 (HR, 0.65 [95% CI, 0.44–0.96]; *P* = 0.029)	41.3 vs. 28.9 (HR, 0.63 [95% CI, 0.39–1.02]; *P =* 0.058)	63.6 vs. 60.5
PRIME (65)	259 vs. 253	Panitumumab + FOLFOX vs. FOLFOX	10.1 vs. 7.9 (HR, 0.72 [95% CI, 0.58–0.90]; *P* = 0.004)	26.0 vs. 20.2 (HR, 0.78 [95% CI, 0.62–0.99]; *P =* 0.04)	Not reported for the *RAS* wt population
PLANET (87)	27 vs. 26	Panitumumab + FOLFOX vs. panitumumab + FOLFIRI	13 vs. 15 (HR, 0.7, 95% CI, 0.4–1.3; *P =* 0.307)	39 vs. 49 (HR, 0.9 [95% CI, 0.4–1.9]; *P =* 0.824)	78 vs. 73 (*P =* 0.691)
VOLFI (88)	63 vs. 33	Panitumumab + mFOLFOXIRI vs. FOLFOXIRI	10.8 vs. 10.5 (HR, 1.11, 95% CI, 0.69–1.75; *P =* 0.6634)	NA	85.7% vs. 60.6% (OR, 3.90 [95% CI, 1.44–10.52]; *P =* 0.0096)

*CALGB, Cancer and Leukemia Group B; CRYSTAL, Cetuximab Combined With Irinotecan in First-Line Therapy for Metastatic Colorectal Cancer; FIRE-3, FOLFIRI Plus Cetuximab vs. FOLFIRI Plus Bevacizumab as First-Line Treatment For Patients With Metastatic Colorectal Cancer; FOLFIRI, leucovorin, fluorouracil, and irinotecan; FOLFOX, leucovorin, fluorouracil, and oxaliplatin; FOLFOXIRI, leucovorin, fluorouracil, oxaliplatin, and irinotecan; HR, hazard ratio; mCRC, metastatic colorectal cancer; mFOLFOXIRI, modified FOLFOXIRI; NA, not applicable; OPUS, Oxaliplatin and Cetuximab in First-Line Treatment of Metastatic Colorectal Cancer; PEAK, Panitumumab Efficacy in Combination With mFOLFOX6 Against Bevacizumab Plus mFOLOFOX6 in mCRC Subjects With Wild-Type KRAS Tumors; PFS, progression-free survival; PRIME, Panitumumab Randomized Trial in Combination With Chemotherapy for Metastatic Colorectal Cancer to Determine Efficacy; OR, odds ratio; ORR, objective response rate; OS, overall survival*.

In recent years, primary tumor location has gained importance as another characteristic of mCRC that impacts patient prognosis and treatment decision making. Primary tumor location (right vs. left, or proximal vs. distal, respectively) has been demonstrated to have significant implications for patient survival and response to available therapies (93). Specifically, patients diagnosed with left-sided tumors have appeared to have better responses with anti-EGFR therapy than with anti-VEGF therapy, with the bulk of tumor location subgroup analysis evidence coming from the available cetuximab-based phase 3 trials. In contrast, patients with right-sided tumors have appeared to derive less benefit from therapy in general (80, 94). In the populations of patients with *RAS* wt left-sided primary tumors in the CALGB/SWOG 80405 and FIRE-3 trials, the median OS approached 40 months with cetuximab plus chemotherapy (FOLFIRI or FOLFOX in CALGB/SWOG 80405 and FOLFIRI in FIRE-3) (80, 94). Indeed, a small retrospective study by Sagawa et al. demonstrated a median OS of over 50 months with cetuximab-based treatment in patients with *RAS* wt left-sided tumors (95). Furthermore, improvements in OS with cetuximab-based treatment were statistically significant compared with bevacizumab-based treatment in the population with *RAS* wt left-sided tumors (80, 94, 95). Efficacy data for first-line panitumumab- vs. bevacizumab-based treatment in *RAS* wt left-sided mCRC are available only from the phase 2 PEAK study, in which OS trended toward improvement with panitumumab; however, the results did not reach statistical significance (96). Although ~86% of the currently published data for first-line studies of anti-EGFR agents vs. bevacizumab in left-sided tumors come from cetuximab trials, studies suggest similar results with either cetuximab or panitumumab compared with bevacizumab in patients with *RAS* wt, left-sided mCRC.

Although patients with right-sided tumors consistently had worse prognoses than patients with left-sided tumors, they may still derive tumor shrinkage benefits with anti-EGFR-mAb-based treatment, according to a meta-analysis by Wang et al. (including the CRYSTAL, TAILOR, PRIME, and 20050181 trials) that demonstrated that anti-EGFR-mAb-based treatment significantly improves response rates and PFS in patients with *RAS* wt mCRC, independent of primary tumor location (97). Additionally, a meta-analysis by Arnold et al. (including the CRYSTAL, FIRE-3, CALGB 80405, PRIME, PEAK, and 20050181 studies) confirmed the prognostic value of primary tumor location and demonstrated that patients with left-sided tumors significantly benefited from an anti-EGFR antibody plus chemotherapy vs. chemotherapy with or without bevacizumab. For patients with right-sided disease, there was no significant benefit in OS or PFS; however, an analysis of ORR showed that an anti-EGFR plus chemotherapy doublet can be a treatment option when cytoreduction is the goal (68). The findings of both meta-analyses support the preferential utilization of an anti-EGFR mAb plus chemotherapy in patients with *RAS* wt, left-sided mCRC, with most of the data being extracted from cetuximab-based trials. Although patients with right-sided tumors tended to derive limited benefit from available therapy, a pooled analysis of prospective trials showed that some proportion of patients with right-sided tumors could respond to cetuximab, suggesting that some patients with right-sided disease may benefit from an anti-EGFR agent plus chemotherapy as an initial treatment (98).

Although cetuximab and panitumumab have not been compared directly in first- or second-line mCRC, a limited number of phase 2 studies exist for each that had comparable trial designs. A phase 2 trial by Carrato et al. evaluated the efficacy of second-line panitumumab plus irinotecan in patients with *KRAS* wt mCRC who had received either 5-FU, oxaliplatin, or irinotecan in the first line. Panitumumab plus irinotecan yielded a PFS and OS of 4.5 and 15.1 months, respectively, and an ORR of 23%. The outcomes observed by Hong et al. with second-line cetuximab plus irinotecan, also in patients with *KRAS* wt disease, were a median PFS and OS of 8.3 and 18.3 months, respectively, and an ORR of 45% (99, 100). The only randomized, phase 3 trial to compare cetuximab and panitumumab directly was ASPECCT, which confirmed the non-inferiority of panitumumab compared with cetuximab as a monotherapy in the third- and later-line setting in patients with *KRAS* wt mCRC. Results of the *RAS* wt subset of the ASPECCT study are still pending (101). In the final analysis, median PFS and OS were 4.1 vs. 4.4 months and 10.4 vs. 10.0 months with panitumumab vs. cetuximab, respectively. The ORR was 22.0% with panitumumab and 19.8% with cetuximab. ASPECCT was a non-inferiority trial (rather than a superiority trial), but a trial powered to investigate efficacy differences between cetuximab and panitumumab in colorectal cancer had not been conducted at the time of this article. Therefore, the results from ASPECCT might not be extrapolated to earlier lines of therapy and to treatment in combination with chemotherapy. One other noteworthy study, the phase 2, randomized WJOG6510G trial, compared cetuximab plus irinotecan and panitumumab plus irinotecan in patients with *KRAS* wt mCRC in whom 5-FU–, oxaliplatin-, and irinotecan-based therapy had previously failed. The results suggested non-inferiority of panitumumab plus irinotecan compared with cetuximab plus irinotecan in this setting (102). Additional third- and further-line studies of cetuximab or panitumumab monotherapy or in combination with irinotecan are difficult to compare directly because many of the early trials with cetuximab were conducted prior to the discovery of the *KRAS* mutation biomarker, and therefore enrollment was determined by EGFR expression status only (103–110). However, the phase 3 CO.17 trial demonstrated how mutation status of the *KRAS* gene was associated with OS in mCRC patients treated with cetuximab after prior chemotherapy (111). More recently, a retrospective analysis of the EPIC study demonstrated that post-study cetuximab was associated with improved OS in the *RAS* wt population (112).

One final difference in clinical efficacy that has been observed between cetuximab and panitumumab concerns the effect of prior bevacizumab treatment on response to subsequent anti-EGFR therapy. Recent evidence has suggested that prior bevacizumab therapy, if administered within a certain time interval of initiation of anti-EGFR therapy, can compromise responsiveness to cetuximab but not to panitumumab (101, 113–117). These findings not only underline the fact that the two mAbs are non-interchangeable, but they also have implications in treatment sequencing—namely, that in order to maximize the potential number of therapeutic lines of treatment, cetuximab should be administered prior to bevacizumab.

## Safety Findings With Cetuximab and Panitumumab in Colorectal Cancer

The rates of grade 3/4 adverse events (AEs) considered related to anti-EGFR therapy in patients treated with first-line anti-EGFR plus chemotherapy are presented in Table 4A. Additionally, the rates of grade 3/4 AEs from the third-line head-to-head ASPECCT trial are presented in Table 4B.

**Table 4A:** Comparison of cetuximab- and panitumumab-associated grade 3/4 adverse events: evidence from **(A)** first-line and **(B)** third-line phase 3 trials.

**Adverse event (%)**	**Treatment regimen**		
	***RAS* wt**	***RAS* wt**	*KRAS* wt^*^
	**CRYSTAL (66) (cetuximab + FOLFIRI)**	**TAILOR (69) (cetuximab + FOLFOX4)**	**PRIME (65, 118) (panitumumab + FOLFOX4)**
Any AE	81	94	57 with grade 3, 28 with grade 4^*^
Diarrhea	15	6	18
Hypomagnesemia	NR	8	7
Infusion-related reactions	2	10	<1
Neurotoxicity	NR	NR	16
Skin reactions	21	26	37
Acne-like rash	17	24	NR

* *Data shown for PRIME, any AE, is from a RAS wt analysis. All other AE data shown for PRIME are from the KRAS wt population*.

*AE, adverse event; CRC, colorectal cancer; CRYSTAL, Cetuximab Combined With Irinotecan in First-Line Therapy for Metastatic Colorectal Cancer; FOLFIRI, leucovorin, fluorouracil, and irinotecan; FOLFOX, leucovorin, fluorouracil, and oxaliplatin; PRIME, Panitumumab Randomized Trial in Combination With Chemotherapy for Metastatic Colorectal Cancer to Determine Efficacy; wt, wild type*.

**Table 4B:** Evidence from the phase 3, head-to-head ASPECCT trial in 3L *KRAS* wt mCRC patients (101).

**Adverse event (%)**	**Treatment**	
	**Cetuximab**	**Panitumumab**
Any AE	494 (98)	485 (98)
**Grade 3/Grade 4 AEs**		
Diarrhea	9 (2)/0	7 (1)/3 (1)
Hypomagnesemia	10 (2)/3 (<1)	26 (5)/9 (2)
Infusion-related reactions	5 (1)/4 (<1)	1 (<0·5)/0
Neurotoxicity	Not reported	Not reported
Skin reactions	48 (10)/0	60 (12)/2 (<0·5)
Acne-like rash	14 (3)/0	17 (3)/0

Although a direct comparison is confounded by the lack of AE rates for *RAS* wt patients in PRIME (the PRIME trial did not present rates of individual AEs for the *RAS* subgroup), the addition of cetuximab to chemotherapy was associated with an increased incidence of grade 3/4 infusion-related reactions, whereas the addition of panitumumab exacerbated the incidence of grade 3/4 diarrhea (65, 66, 69, 118). A meta analysis by Petrelli et al. concluded that while cetuximab and panitumumab have a similar burden of overall toxicity in terms of severe AEs, the individual safety profiles are distinct. Panitumumab was associated with a higher rates of grade 3/4 skin toxicities, hypomagnesemia, fatal AEs, and treatment discontinuations, while cetuximab was associated with a higher rates of skin rash, infusion reactions, and gastrointestinal toxicity (119). As noted in Petrelli et al., the third-line, anti-EGFR monotherapy trial ASPECCT also identified increased rates of grade 3/4 hypomagnesemia and decreased rates of infusion-related reactions with panitumumab compared with cetuximab (101). Finally, whereas the CRYSTAL and TAILOR trials reported no treatment-related grade 3/4 neurotoxicity occurring at a rate of ≥5% frequency in either arm, a rate of 16% was reported in the patient population of the PRIME trial (118). Petrelli et al. similarly identified a higher rate of grade 3/4 neurotoxicity in panitumumab trials than in cetuximab trials (119). The reasons for the increased incidence of (likely oxaliplatin-related) neurotoxicity (120) in panitumumab trials remain unknown.

Regarding chemotherapy backbones for the two mAbs, the selection of FOLFIRI vs. FOLFOX for first-line treatment can depend on which toxicity profile is likely to be more tolerable for the patient in question, because the two regimens are considered to have similar activities in mCRC (2). Therefore, differences in the toxicity profiles between the two chemotherapy backbones in combination with panitumumab vs. cetuximab are of substantial clinical relevance during treatment selection. However, it is worth noting that a meta-analysis by Teng et al. found a slight improvement in time to progression, and thus in OS, with FOLFIRI followed by FOLFOX compared with the reverse sequence (121). This finding reinforces the importance of treatment sequencing and how the differential findings with cetuximab and panitumumab can be applied, namely, that cetuximab has been shown to pair well with either FOLFOX or FOLFIRI vs. FOLFOX or FOLFIRI alone, whereas all available phase 3 data for panitumumab efficacy in first-line mCRC are in combination with FOLFOX. Notably, there are several small studies, although without comparator arms, that have provided evidence for the activity of panitumumab in combination with FOLFIRI in mCRC (87, 90).

## Efficacy With Cetuximab and Panitumumab in Head and Neck Cancer

As previously mentioned, cetuximab has been approved for use in combination with radiotherapy in locally advanced SCCHN (LA SCCHN) and in combination with platinum and 5-FU, followed by cetuximab maintenance, for recurrent and/or metastatic SCCHN (R/M SCCHN) (122, 123). Panitumumab has been investigated in combination with radiotherapy in LA SCCHN but has failed to improve upon the current standard-of-care chemoradiotherapy treatment (124, 125), and it did not demonstrate a significant improvement in OS when added to platinum plus 5-FU chemotherapy in the R/M setting (126). A caveat is that panitumumab maintenance was optional in the SPECTRUM trial, following panitumumab plus platinum and 5-FU in patients with first-line R/M SCCHN, whereas cetuximab maintenance therapy in the EXTREME trial was given to all patients who achieved stable disease or a response during combination treatment (126, 127). Therefore, we are unable to directly compare the two agents in the SCCHN setting (Tables 5A,B). What can be said with certainty is that cetuximab is highly active in SCCHN, and proposed explanations include the increased potential contribution of cetuximab's immune actions in this tumor type, given the predominance of EGFR-overexpressing cells and immunologic sensitivity in head and neck tumors (25). Specifically, cetuximab's stimulation of ADCC and other immunostimulatory activities (DC maturation, T-cell recruitment to the tumor, increased antigen presentation, and cytotoxic T-cell priming) are dependent on cetuximab's simultaneous binding of the EGFR and the CD16 receptor on NK cells (25). Indeed, evidence has suggested the link between high baseline ADCC and EGFR overexpression and better outcomes with cetuximab plus radiotherapy but not with chemoradiotherapy (55). Thus, while it is difficult to prove the clinical impact of cetuximab-driven immunostimulation on tumor cell death, tumor shrinkage, and disease control, a wealth of evidence suggests that it is, in fact, a contributing factor to cetuximab's antitumor activity in SCCHN (25), and it may be the key differentiating aspect between cetuximab and panitumumab in head and neck cancer.

**Table 5A:** Clinical impact of cetuximab and panitumumab in LA SCCHN.

**Study**	**Treatment regimen**	**Patients, *n***	**LRC rate** **(2 years)**	**OS rate (2 years)**	**Safety findings**
IMCL-9815 (Bonner trial) (128)	Radiotherapy vs. radiotherapy + cetuximab	213 vs. 211	41 vs. 50%	55 vs. 62%	Grade 3–5 mucositis (52 vs. 56%), acneiform rash (1 vs. 17%), radiation dermatitis (18 vs. 23%), weight loss (7 vs. 11%), xerostomia (3 vs. 5%), dysphagia (30 vs. 26%), asthenia (5 vs. 4%), constipation (5 vs. 5%), pain (7 vs. 6%), and dehydration (8 vs. 6%)
CONCERT-2 (124)	Chemoradiotherapy vs. radiotherapy + panitumumab	61 vs. 90	61 vs. 51%	71 vs. 63%	Grade 3/4 mucositis (40 vs. 42%), dysphagia (32 vs. 40%), radiation skin injury (11 vs. 24%). Serious AEs were more frequent in the chemoradiotherapy arm (40 vs. 34%)
Siu et al. (125)	Chemoradiotherapy vs. radiotherapy + panitumumab	156 vs. 159	73 vs. 76% (2-year PFS rate)	85 vs. 88%	Grade ≥ 3 non-hematologic AEs occurred at rates of 88 vs. 91%, respectively

**Table 5B:** Clinical impact of cetuximab and panitumumab in R/M SCCHN.

**Study**	**Treatment regimen**	**Patients, *n***	**Median PFS, months**	**Median OS, months**	**Safety findings**
EXTREME (127)	Cisplatin/carboplatin + 5-FU + cetuximab → maintenance cetuximab vs. Cisplatin/carboplatin + 5-FU	222 vs. 220	5.6 vs. 3.3 (HR, 0.54 [95% CI, 0.43–0.67]; *P* < 0.001)	10.1 vs. 7.4 (HR, 0.80 [95% CI, 0.64–0.99]; *P =* 0.04)	Grade 3/4 neutropenia (22 vs. 23%), anemia (13 vs. 19%), thrombocytopenia (11 vs. 11%), skin reactions (9 vs. < 1%)
SPECTRUM (126)	Cisplatin/carboplatin + 5-FU + panitumumab → maintenance panitumumab q3w (optional) vs. Cisplatin/carboplatin + 5-FU	327 vs. 330	5.8 vs. 4.6 (HR, 0.78 [95% CI, 0.659–0.922]; *P =* 0.0036)	11.1 vs. 9.0 (HR, 0.873 [95% CI, 0.729–1.046]; *P =* 0.1403)	Grade 3/4 skin or eye toxicity (19%), diarrhea (5%), hypomagnesemia (12%), hypokalemia (10%), and dehydration (5%) were more frequent in the panitumumab arm vs. control. 4% treatment-related deaths occurred in the panitumumab arm

*5-FU, fluorouracil; AE, adverse event; CONCERT-2, Concomitant Chemotherapy and/or EGFR Inhibition With Radiation Therapy; EXTREME, Erbitux in First-Line Treatment of Recurrent or Metastatic Head and Neck Cancer; HR, hazard ratio; LA, locally advanced; LRC, locoregional control; OS, overall survival; PFS, progression-free survival; q3w, every 3 weeks; R/M, recurrent and/or metastatic; SCCHN, squamous cell carcinoma of the head and neck; SPECTRUM, Study of Panitumumab Efficacy in Patients With Recurrent and/or Metastatic Head and Neck Cancer*.

## Potential of Anti-EGFR mAbs in Combination With Immunotherapy Regimens

Cetuximab and panitumumab behave differently, despite their therapeutic targeting of the same receptor; thus, available clinical data for one should not be applied to the other. Looking to the future in mCRC treatment, emerging immunotherapies have yet to demonstrate paradigm-shifting clinical activity in mismatch repair–proficient mCRC (129), suggesting that the way forward will continue to be combinatorial, including chemotherapy elements. In this respect, irinotecan's and oxaliplatin's synergistic effects with cetuximab (130–132) and possible differences from a treatment-sequencing standpoint suggest that cetuximab plus either FOLFIRI or FOLFOX is a suitable combination partner for checkpoint inhibitors and other immunotherapies. For example, cetuximab induces NK cell–mediated ADCC, resulting in increased immunogenic cell death, and cetuximab-treated cells have been shown to be more susceptible to phagocytosis by DCs. In the same study by Pozzi et al., even measurable immunogenic cell death occurred when CRC cell lines and mouse CRC models were co-treated with cetuximab plus FOLFIRI (31). Similarly, oxaliplatin has been shown to have some immunostimulatory properties, including immunogenic cell death (133–136) and the ability to prime tumors for checkpoint blockade in preclinical models (11, 136, 137). Cetuximab's known immune actions, including increasing immune infiltration and immune visibility of the tumor, suggest that it will be the more potent combination partner for either irinotecan- or oxaliplatin-based therapy, to which checkpoint inhibitors may theoretically be added to increase the immune antitumor response.

## Conclusions and Future Directions for Cetuximab and Panitumumab

Cetuximab and panitumumab are both currently used to treat *RAS* wt mCRC. Clinical data for panitumumab in combination with chemotherapy is mostly limited to FOLFOX in the first-line setting, whereas cetuximab has demonstrated efficacy and safety in phase 3 first-line trials with both FOLFOX and FOLFIRI. Additionally, their combinability with FOLFIRI and known activity following prior bevacizumab treatment may have implications for optimal treatment sequencing in the continuum of care for mCRC.

Aside from the fact that panitumumab is a human mAb and cetuximab is a mouse/human chimeric mAb, the two anti-EGFR agents are composed of different IgG isotypes. Because cetuximab is an IgG1 mAb, it has additional immunogenic activity not demonstrated by panitumumab (IgG2). Cetuximab, unlike panitumumab, can prime the tumor microenvironment for an immune attack by enabling multiple processes, including ADCC and activation of innate and adaptive immune effector cells. Interestingly, both cetuximab and panitumumab improve outcomes in CRC. Despite extensive immune system activation induced by cetuximab, residual tumor-associated cells can prevent the final attack of cytotoxic T cells on the tumor by upregulation of PD-1, PD-L1, and CTLA-4 on their surface or by releasing cytokines such as TGF-β or chemokines such as CXCL12, which inactivate effector cells (25). Whether cetuximab will be clinically superior to panitumumab in the immunotherapy era remains to be determined by future clinical trials employing immune checkpoint inhibitors, which may complement the “immune priming” activity of cetuximab (and chemotherapy). We eagerly anticipate upcoming results from future and ongoing clinical trials.

## Author Contributions

All authors contributed equally to the conception of the intellectual content, interpretation of the data, and writing of the manuscript. All authors also reviewed any revisions that were made and provided their final approval of the manuscript.

### Conflict of Interest Statement

JG-F has had an advisory role and has received honoraria for talks from Amgen, Bayer, Sanofi, Merck Healthcare KGaA, Roche, Servier, Eli Lilly, Novartis, Pfizer, BMS, MSD, and AstraZeneca. YS has received honoraria for talks from Taiho Pharmaceutical, Chugai Pharmaceutical, Yakult Honsha, Takeda, Merck Serono, Bayer Yakuhin, and Sanofi. DA has had an advisory role and has received honoraria from Merck Healthcare KGaA, Teva, and Bayer. ZW has received consultation fees and honoraria from EMD Serono, Lilly, Genentech, and Novartis. PR and PW are employees of Merck Healthcare KGaA, Darmstadt, Germany. SS has had an advisory role and has received honoraria for talks from Amgen, Bayer, Eli Lilly, Merck Healthcare KGaA, Roche, Sanofi, and Takeda.
